# Effects of *Ganoderma lucidum* Powder on the Growth Performance, Immune Organ Weights, Cecal Microbiology, Serum Immunoglobulins, and Tibia Minerals of Broiler Chickens

**DOI:** 10.3390/vetsci11120675

**Published:** 2024-12-22

**Authors:** Arazay Avain, Md. Abul Kalam Azad, Yaneisy García, Yanelys García, Yordan Martínez

**Affiliations:** 1 Sustainable Tropical Agriculture Master Program, Zamorano University, Valle de Yeguare, San Antonio de Oriente P.O. Box 93, Francisco Morazán, Honduras; 2 CAS Key Laboratory of Agro-Ecological Processes in Subtropical Region, Hunan Provincial Key Laboratory of Animal Nutritional Physiology and Metabolic Processes, Institute of Subtropical Agriculture, Chinese Academy of Sciences, Changsha 410125, China; 3 Department of Monogastric Animals, Institute of Animal Science, Central Highway km 47 ½, San José de las Lajas C.P. 32700, Mayabeque, Cuba; 4 Faculty of Veterinary Medicine, University of Fondwa, Léogâne 6320, Haiti

**Keywords:** growing chickens, fungus powder, productivity, immunology, bacteria

## Abstract

A total of 640 one-day-old Cobb 500 MV × Cobb 500 FF mixed broilers were randomly assigned to one of four experimental treatments with four replicates per treatment and 40 birds per replicate for 32 days. The treatments consisted of a basal diet (control group), basal diet + 0.02% zinc bacitracin (AGP group), basal diet + 0.2% *G. lucidum* powder (GLP; 0.2% GLP group), and basal diet + 0.3% GLP (0.3% GLP group). The results showed that dietary 0.2% GLP supplementation increased body weight compared to the control and 0.3% GLP groups, and decreased feed conversion ratio (FCR) compared to the control group, during 19–32 days (*p* < 0.05). The feed intake was lower (*p* < 0.05) in both dietary GLP supplementation groups and the AGP group during 1–8 and 1–32 days compared to the control group. Additionally, the FCR was lower in the dietary GLP supplementation group (0.2%) and the AGP group (*p* < 0.05) compared to the control group. Moreover, the caeca of broiler chickens in the AGP and 0.2% GLP groups had a higher abundance of lactic acid bacteria (LAB). Supplementation of feed additives (AGP and GLP) increased the relative weight of the thymus, with no effect on the bursa of Fabricius and spleen. However, AGP supplementation decreased the serum IgM concentration, while supplementing a higher dose of GLP (0.3%) increased the ash content in the tibia. The findings indicate that 0.2% GLP is the recommended supplementation dose as a natural growth promoter to replace AGP in apparently normal chickens.

## Simple Summary

*Ganoderma lucidum* fungi are a rich source of several secondary metabolites, such as triterpenes, saponins, steroids, alkaloids, ganadic acid, and β-glucans. Although some Asian countries recommend their use in humans, little information existson alternatives to antibiotic growth promoters (AGP) in animals. Thus, this study aimed to demonstrate that *G. lucidum* powder (GLP) could positively respond to body weight gain, feed intake, and feed conversion ratio in broilers up to 32 days old. Moreover, GLP supplementation modulates intestinal microbiology by increasing the abundance of lactic acid bacteria without changing the concentrations of immunoglobulins; however, the AGP group had a decreased serum concentration of IgM. The higher level of GLP supplementation seemed to improve tibial mineralization; however, a broader mineral profile needs further investigation. These findings will contribute to the use of new natural products rich in beneficial chemical compounds as an alternative to AGPs in broiler production.

## 1. Introduction

The poultry industry is one of the most important economic sources in the agricultural sector, due to its growing development concerning other food production systems of animal origin [1]. The intensive production system is associated with several stressors for broiler chickens that cause intestinal microbiota dysbiosis due to the development of pathogenic microorganisms, immunosuppression, and inefficient feed conversion [2]. To overcome these effects, antibiotic growth promoters (AGPs) have been widely used since the 1950s [3]. However, the continuous use of antibiotics, even at a subtherapeutic level, increases the bioaccumulation of antibiotic resistance in muscles, cross-resistance to other microorganisms, and the possibility of transportation of antibiotic residues in food. Furthermore, the misuse or overuse of antibiotics causes ecological balance dysbiosis of the gastrointestinal microbiota, predisposing animals to contract different diseases [4]. These problems have raised a growing interest in reducing and even prohibiting the application of antibiotics in poultry production systems [5].

With the ban on the use of AGP, many producers and researchers are looking for AGP-alternative feed additives, especially to achieve effective control of mortality and improve feed conversion rates in the poultry industry [6]. Among the possible antibiotic alternatives in poultry production, prebiotics, probiotics, symbiotics, postbiotics, phytobiotics, and acidifiers have been extensively studied [7,8]. Higher basidiomycete fungi constitute a natural option for AGP alternatives. These fungi have a high nutritional value, with rich sources of essential minerals and vitamins, as well as unsaturated fatty acids. Beyond their nutritional value, fungi are also rich sources of secondary metabolites with nutraceutical properties, which have gained significant interest in nutritional strategies in animal nutrition [9,10]. Recent studies have demonstrated that different secondary metabolites produced by fungi could improve the lipid metabolism and gastrointestinal health of poultry [11,12,13]. Such bioactive compounds have the potential for use as natural feed additives, thereby improving the production performance of poultry [1]. *G. lucidum* has been identified as a rich source of triterpenes, saponins, steroids, alkaloids, ganodic acid, and β-glucans, which are used by an organism’s immune system to detoxify the organism [14]. Several previous studies have shown that *G. lucidum* extracts could improve the feed conversion and viability of broiler chickens [15]. On the other hand, this medicinal fungus can also promote immune function, due to the production and excretion of interferon by host cells and the action of macrophages and T lymphocytes [1,16]. Currently, few studies have evaluated the effects of *G. lucidum* compared with other AGPs in broiler production. Thus, further research is necessary to achieve total replacement of AGPs, especially with natural resources rich in beneficial secondary metabolites. Therefore, the present study aimed to evaluate the effects of dietary *G. lucidum* powder (GLP) supplementation on the growth performance, immune organ weights, cecal microbiology, serum immunoglobulins, and tibia minerals of broiler chickens.

## 2. Materials and Methods

### 2.1. Location of the Study

The study was carried out at the Poultry Research and Teaching Center of Zamorano University, Tegucigalpa, San Antonio de Oriente, Francisco Morazán, Honduras.

### 2.2. Collection and Preparation of G. lucidum

The fungus (*G. lucidum*) was collected from the Uyuca Ecological Reserve, San Antonio de Oriente, Honduras. Dried *G. lucidum* samples were placed in a fiber bag to prevent moisture absorption during transportation to the laboratory. The fungus samples were cleaned with distilled water to remove impurities, placed in paper bags, and dried in an oven for three days at 65 °C. The samples were ground with a knife mill (Thomas Wiley, Model 4; Swedesboro, NJ, USA) and stored in sealed plastic bags at room temperature until further use. The GLP contained 10.60% dry matter, 9.81% crude protein, 2.83% ash, 0.32% calcium, 0.19% magnesium, 0.10% phosphorus, 0.49% potassium, 5.01 mg/kg copper, and 32.67% zinc.

### 2.3. Experimental Design, Animals, and Treatments

A total of 640 one-day-old Cobb500™ MV × FF genetic line broiler chickens (males and females) were randomly allocated into four treatment groups. Each treatment group consisted of four replicates and 40 chickens per replicate. The experimental treatment groups included the control group (fed with an antibiotic-free basal diet), AGP group (0.02% zinc bacitracin with the basal diet), 0.2% GLP group (0.2% GLP supplementation with the basal diet), and 0.3% GLP group (0.3% GLP supplementation with the basal diet). The feeding trial lasted 32 days. The composition and nutrient levels of the experimental diets for this study were based on the standard nutritional requirements of the genetic line (Table 1).

### 2.4. Experimental Conditions

The birds were housed in a deep wooden chip bed (11 birds/m^2^). Feed and water were available ad libitum with a hopper feeder and water nipple. The temperature and ventilation inside the rearing house were controlled by gas brooders, curtain management, and fans. The barn was disinfected (with 5% quaternary ammonium), as per the environmental quality standards of the Poultry Research and Training Center Protocol, 24 h before the experimental chicks entered the area. No medications or therapeutic veterinary care were used throughout the experimental trial. The birds were vaccinated against Newcastle, Gumboro, and infectious bronchitis.

### 2.5. Growth Performance

The initial and final body weight (BW) of the birds per replicate was measured using a Mettler Toledo IBOA224-15NP (Changzhou, China) industrial scale with an accuracy of ± 2.0 g. The feed intake and feed refusal per replicate were recorded weekly to calculate the feed intake (FI) and feed conversion ratio (FCR). Viability was assessed according to the number of living animals remaining among those existing at the beginning of the experiment. The growth performance indicators, including the BW, FI, FCR, and viability of the broiler chickens, were determined for each experimental phase (1–8, 9–18, and 19–32 days of the trial).

### 2.6. Relative Weights of Immune Organs

On 32 days of the trial, 20 broiler chickens (10 males and 10 females) were selected for sampling from each experimental treatment group after weighing (Mettler Toledo IBOA224-15NP; Changzhou, China). After 8 h fasting, selected broiler chickens were slaughtered by a certified veterinarian using the mechanical cervical dislocation (stunning) method, and once the birds were unconscious, the exsanguination technique was used. Subsequently, the abdominal cavity was opened, and the immune organs (the bursa of Fabricius, thymus, and spleen) were removed and weighed to calculate the relative organ index.

### 2.7. Cecal Microbiology

The left caeca contents of 8 birds per treatment (4 females and 4 males) were randomly selected, and the mucosa was scraped with a scalpel for microbiological culture. The caeca content of each sample was placed in a sterile centrifuge tube, weighed, and diluted with an aqueous solution of Butterfield’s buffered phosphate at 1:10 (*w*/*v*). The diluted caeca contents were homogenized, and serial dilutions (1/10) were prepared up to 10^5^ dilution. Subsequently, 0.1 mL aliquots of each dilution were taken and spread on plates on a surface of MRS Agar (Neogen Acumedia, Lansing, MI, USA) supplemented with methylene blue (0.016 g/1000 mL), and kept at 37 °C, with a pH of 5.6, for 48 h, in anaerobiosis (Gas Pak System, BBL, Cockeysville, MD, USA). Violet Red Bile Glucose agar plates for *Enterocateriaceae*, and Violet Red Bile Lactose MUG Agar for coliform and *Escherichia coli* counts (Liofilchem, Teramo, Italy), were incubated at 35 °C for 24 h. The abundances of yeast and molds were determined using a Rose-Bengal Choramphenicol Agar (Liofilchem, Taramo, Italy), incubated at 25 °C for five days. Lactic acid bacteria counts were calculated as 10 CFU/g by colony morphology on MRS+MB Agar. Gram staining and catalase tests were conducted for each colony type, as described previously [1]. An LX400 light microscope (Fremont, CA, USA) was used for the morphological characterization of the bacterial colonies.

### 2.8. Immunoglobulin Concentration

On day 32, 5 mL of blood samples were collected from the jugular vein of 8 birds (4 male and 4 female) per treatment. The blood samples were placed in 2 mL tubes, then sodium heparin was added at a ratio of 2:1, and they were stored at −15 °C. The protocols for the quantification of the immunoglobulins IgG, IgA, and IgM were followed according to the manufacturers’ instructions labeled on the ELISA kits (Nanjing Jiancheng Bioengineering Institute, Nanjing, China) and according to the user manual at the certified Clinical Analysis Laboratory of Paredes y Asociados, Tegucigalpa, Honduras.

### 2.9. Tibia Minerals

After slaughter, 8 tibia (from 4 females and 4 males) were randomly selected per treatment. The samples were stored at −4 °C and transferred to the Chemical Laboratory of Zamorano University to determine their ash and Ca contents according to AOAC Standard 965.09, and their P content, by the colorimetric method.

### 2.10. Statistical Analysis

The data were analyzed by one-way analysis of variance (ANOVA) in a completely randomized design. The Kolmogorov–Smirnov test verified the normality of the data, and for the uniformity of the variance, the Bartlett test was performed. Duncan’s test was conducted to determine the differences between the groups when necessary. Viability was calculated by comparing proportions. All the analyses were performed with the statistical software SPSS version 25.1 (SPSS Inc., Chicago, IL, USA).

## 3. Results

### 3.1. Effects of G. lucidum Powder Supplementation on the Growth Performance of Broiler Chickens

The effects of GLP supplementation on the production performance of broiler chickens are presented in Table 2. During the starter stage (1–8 days), both the dietary GLP supplementation groups and the AGP supplementation group had a decreased (*p* < 0.05) FI compared to the control group, whereas the dietary 0.2% GLP supplementation group and the AGP supplementation group had a decreased (*p* < 0.05) FCR compared to the control group. During the grower stage (9–18 days), the 0.2% GLP group had a decreased (*p* < 0.05) FI compared to the control and AGP group. Additionally, the FCR was lower (*p* < 0.05) in the 0.2% GLP group in relation to the other three groups. However, at both productive stages (starter and grower), GLP supplementation did not change the BW and viability of the broiler chickens (*p* > 0.05). From the analysis of Table 2, the FCR at the finishing stage (19–32 days) in both dietary GLP supplementation groups and the AGP group was improved compared to the control group. However, this improvement was only significant (*p* < 0.05) for the AGP group and the dietary GLP supplementation (0.2%) group. The FCR in the dietary GLP supplementation (0.3%) group was statistically similar to all the other groups. Considering the entire experimental period (1–32 days), viability did not differ significantly between treatments (*p* > 0.05); however, both the dietary GLP supplementation groups and the AGP group had a decreased (*p* < 0.05) FI, and the dietary 0.2% GLP supplementation group a decreased (*p* > 0.05) FCR, compared to the control group.

### 3.2. Effects of G. lucidum Powder Supplementation on the Cecal Microbiology of Broiler Chickens

Dietary GLP and AGP supplementation did not change (*p* > 0.05) the quantification of total coliforms, *E. coli*, *Enterobacteriaceae*, pH, fungi, and yeast in the caeca of broiler chickens. The abundance of lactic acid bacteria in the caeca of broiler chickens was increased (*p* < 0.05) in the AGP and 0.2% GLP groups compared to the control group (Table 3).

### 3.3. Effects of G. lucidum Powder on the Relative Weight of Immune Organs of Broiler Chickens

The effects of dietary GLP and AGP on the relative weights of immune organs in broiler chickens are presented in Table 4. The relative weight of the thymus of broilers in both the dietary GLP supplementation groups and the AGP group was increased (*p* < 0.05) compared to the control group. The other immune organs (bursa of Fabricius and spleen) were not affected (*p* > 0.05) by dietary GLP and AGP supplementation.

### 3.4. Effects of G. lucidum Powder on Immunoglobulin Concentrations of Broiler Chickens

The AGP group had a decreased (*p* < 0.05) serum IgM concentration compared to the control and 0.3% GLP groups. The serum IgM concentration in the dietary GLP supplementation (0.2%) group was statistically similar to all the other groups (*p* > 0.05). Moreover, supplementation in both the GLP supplementation groups and the AGP group did not significantly change the serum IgG and IgA concentrations in broiler chickens (*p* > 0.05; Table 5).

### 3.5. Effects of G. lucidum Powder on Tibia Minerals of Broiler Chickens

Dietary 0.3% GLP supplementation increased (*p* < 0.05) the ash content of the tibia of broiler chickens compared to the other experimental groups. In addition, the Ca and P contents of the tibia remained unchanged by AGP and GLP supplementation (*p* > 0.05; Table 6).

## 4. Discussion

This study demonstrates that the medicinal mushroom *G. lucidum* can positively influence animal performance compared to a common antibiotic growth promoter in broiler production. The scientific hypothesis is that the efficacy of *G. lucidum* is attributed to its diversity in its chemical composition; triterpenoids and other bioactive compounds are its main secondary metabolites [17,18]. Thus, various active components of *G. lucidum* allow its use as a nutraceutical feed additive in poultry diets. The use of this type of nutraceutical feed additive may contribute to improved animal health and productive performance.

During the present investigation, the BW, FCR, and viability of broilers demonstrated that the experimental conditions during the 32 days of rearing allowed the animals to efficiently express their genetic potential, as indicated by the genetic line [19]. The findings also demonstrated that GLP supplementation at a low concentration dose (0.2%) did not cause morbidity and mortality in broiler chickens. The animals’ response to feed additives depends on the type and concentration of the additives. Several secondary metabolites, such as tannins, flavonoids, alkaloids, and polyphenols at lower concentrations, positively influence the production performance of the animals [18]. However, a higher concentration of these secondary metabolites can cause adverse effects in broilers, such as intestinal inflammation, decreased nutrient absorption, and diarrheal syndrome [20]. For this reason, it is necessary to determine adequate levels of natural growth promoters that can generate a positive response by modulating the intestinal microflora and immunity of animals [6].

The dietary GLP supplementation groups and the AGP group had a reduced FI during 1–8 and 1–32 days of the trial. However, a decrease in feed intake did not affect the performance of the broiler chickens. Nevertheless, the reduced FI could be related to the secondary metabolites in GLP that can decrease the transit rate of feed chyme in the small intestine, which favors the digestion and absorption of nutrients [21]; this could have translated into better feed efficiency (FCR) in these animals [22]. In this sense, FCR is the main indicator of animal productivity, because it shows the ability of animals to transform feed into body mass. In the present study, dietary supplementation with 0.2% GLP decreased the FCR of broiler chickens throughout the trial compared to the control group, indicating that GLP in the diet allows the optimization of nutrient ingestion according to the digestive physiology of the bird. Thus, in this study, GLP used as a nutraceutical additive showed a growth-promoting effect similar to AGP, which might be due to its anti-inflammatory, antioxidant, and antibacterial properties, and may influence the improved nutrient digestibility and, thus, the productive performance of animals [16,23,24,25].

Moreover, GLP supplementation in the broiler chickens’ diets increased the relative weight of the thymus. Changes in lymphoid organs (thymus, bursa of Fabricius, and spleen) are considered part of the immune response of broilers [26]. Previous studies on GLP and its extract have reported a higher relative weight of the thymus and productive response of broilers, possibly due to hematopoiesis and modulation of the immune response in young animals [1,25]. Thus, it is confirmed that *G. lucidum* has immunostimulant activity in chickens, which is directly associated with secondary metabolites, mainly monosaccharides, disaccharides, and polysaccharides, with an emphasis on β-glucan [27]. Polysaccharides from GLP modulate the immunomodulatory mechanism by promoting macrophage proliferation and the activation of T and B lymphocytes and natural killer cells, which are critical for modulating non-specific immunity [24,28]. It should be noted that this immunomodulatory mechanism has become the premise for other bioactivities, such as anti-inflammatory activity. Therefore, dietary GLP supplementation may improve the immune response of broiler chickens.

Intestinal health directly influences production performance within commercial flocks. A stable microbiota composition is essential for the adequate development of physiological, nutritional, and immunological processes [1]. Regarding the cecal microbiota analysis in the present study, the results indicate that the chemical composition of the diet may influence the stability of the caecal microbiota, which is essential for the intestinal health and productivity of broilers [4,5]. As an effect of dietary treatments (AGP and 0.2% GLP), there was an increase in the LAB counts compared to the control group. The increased LAB count in the AGP group is due to the fact that this type of AGP can modulate the composition of the intestinal microbiota, and thus improve the intestinal health of poultry [6]. However, there is a move towards replacing its use in animal production in the future, due to its consequences for the food and nutritional security of human beings. The increase in LAB counts in the 0.2% GLP group might be related to the effect that the polysaccharides of this fungus may regulate the intestinal microbiota by increasing the relative abundance, of Lactobacilli, among others [27,28,29].

Several previous studies, such as those by Chuang et al. [30] and Chuang et al. [31], have found that *G. lucidum* can modulate the cecal microbial community in normal and challenged with lipopolysaccharide (LPS) broilers by enhancing the abundance of different beneficial bacterial genera, due to its chemical compounds (particularly polysaccharides). Authors have also emphasized that non-digestible carbohydrates, such as oligosaccharides and polysaccharides, are considered compounds with prebiotic potential, which might be associated with changes in bacterial composition. Additionally, supplementation of polysaccharides extracted from *Lentinus edodes*, *Tremella fuciformis*, and *Astragalus gallinaceus* increased the abundance of Bifidobacteria and Lactobacilli in broiler chickens when compared with control and AGP groups [29]. The chemical structure of medicinal herbs does not allow them to be hydrolyzed by digestive enzymes, allowing them to reach the large intestine and be absorbed, serving as a substrate for the colonization of beneficial bacteria [32]. Furthermore, a higher abundance of LAB increases intestinal competitive exclusion, a crucial process in reducing pathogenic bacteria [33]. However, in the present study, no statistical variations were observed in yeast counts, indicating no specific effect of the treatments. It is well known that a higher abundance of LAB can inhibit yeast growth in the caecum due to the higher concentration of organic acids [29,30]. Total coliforms and *E. coli* were not changed by dietary supplementation of GLP in apparently healthy animals, demonstrating that GLP does not favor the growth of pathogenic enterobacteria, which is recurrent in poultry production.

Serum immunoglobulin concentrations reflect the humoral immune status of animals. There are five types of immunoglobulins, of which IgA, IgG, and IgM are the most significant in the immune response of broiler chickens [34]. It should be noted that there are still many inconsistencies in terms of whether the use of alternative additives to AGPs can modify immune activity, because the mechanisms of immunological activity are not completely clarified. In the present study, a decrease in the concentration of IgM was found in the AGP group, suggesting that these synthetic growth-promoting products can depress the humoral immune response. IgM is the main immunoglobulin produced during the primary immune response, although it is also found in secondary responses. IgM is more effective than IgG in complementing activation, virus neutralization, and agglutination [35]. Li et al. [36] found an increase in IgM when broilers’ diets were supplemented with oligosaccharides. However, the concentration of IgM was statistically similar in the GLP-supplemented groups compared with the control group. Additionally, although the concentrations of IgA and IgG did not vary between treatments, Mahfuz et al. [37] observed an increase in serum IgG concentrations in broiler chickens that consumed *Flammulina velutipes* fungus waste, which they attributed to the presence of β-glucans among the secondary metabolites present in the fungus waste. Moreover, Ullah et al. [38] did not find changes in IgA concentrations in a study with *Pleurotus ostreatus*, although IgG and IgM concentrations varied significantly. Therefore, the inconsistencies in these findings may be due to the chemical composition of feed additives and the animal breed, which warrant further investigation.

Little information exists on the effect of nutraceutical additives on the tibia mineralization of broiler chickens. In the present study, changes in ash content were only observed in the 0.3% GLP supplementation group compared to the other three groups. This finding indicates that dietary 0.3% GLP supplementation could improve bone mineralization in broiler chickens, although other mineral contents (Ca and P) did not significantly change among the different groups. According to Wu et al. [39], bone mineralization positively correlates with tibiotarsal index, bone density, and bone fracture resistance. A previous study by Sharif et al. [40] reported that *G. lucidum* has higher concentrations of Li, Mg, Na, Zn, and K compared with four other commercial edible mushroom species, including *Lentinus edodes*, *Hericium erinaceus*, *Volvariella volvacea*, and *Salmalia malabarica*. Therefore, this might be one of the possible reasons why supplementation of a higher dose of *G. lucidum* could encourage higher tibia mineralization. Furthermore, the beneficial secondary metabolites (mainly β-glucans) in *G. lucidum* could benefit gut health and mineral absorption in the tibia [41,42,43]. Javid et al. [44] also reported that a mannan-oligosaccharide-rich additive supplementation exhibited a higher ash content in the tibia of broilers; the authors justified these results by the fact that this natural feed additive can modulate gut microflora and, in turn, improve gut health. Several previous studies have reported inconsistencies in the relationship of ash content with Ca and P content. For instance, Rousseau et al. [45] and Ceylan et al. [46] reported that Ca and P deficiencies in the tibia were related to lower ash content, which caused bone diseases in broilers, although the authors emphasized the age of the animals. Thus, it is noteworthy to evaluate other minerals in the tibia to elucidate the relationship of ash content with other minerals. Notably, the animals in this study did not present bone or locomotion diseases.

## 5. Conclusions

Dietary *Ganoderma lucidum* powder supplementation (mainly 0.2% GLP) promoted body weight at the finisher stage, and it increased the feed efficiency and caecal microbiology of broiler chickens. However, antibiotic growth promoter (0.02% zinc bacitracin) supplementation exhibited immunity-lowering effects by decreasing serum IgM. Moreover, dietary *Ganoderma lucidum* powder supplementation increased the relative weight of the thymus without affecting the other lymphoid organs. Thus, the findings indicate that dietary 0.2% *Ganoderma lucidum* powder supplementation, as a replacement for antibiotic growth promoters, had better impacts on broiler chickens.

## Figures and Tables

**Table 1:** Ingredients and nutritional composition of experimental diets (%, air-dried).

		Experimental Diets	
Ingredients (%)	0–8 Days	9–18 Days	19–32 Days
Corn meal	51.36	56.75	58.53
Soybean meal	39.16	34.27	32.1
Premix ^1^	0.35	0.35	0.35
Sodium chloride	0.23	0.23	0.23
Sodium bicarbonate	0.28	0.28	0.28
African palm oil	4.81	4.6	5.44
Choline chloride	0.05	0.05	0.05
DL-methionine	0.34	0.31	0.28
L-threonine	0.12	0.07	0.03
L-lysine	0.19	0.19	0.14
Calcium carbonate	1.39	1.31	1.16
Monocalcium phosphate	1.50	1.37	1.19
Mycotoxin sequestrant	0.12	0.12	0.12
Exogenous enzymes	0.05	0.05	0.05
Coccidiostat	0.05	0.05	0.05
Nutritional contribution (%)			
ME (kcal/kg)	2975.00	3025.00	3100.00
Crude protein	22.00	20.00	19.00
Lys	1.22	1.12	1.02
Met + Cys	0.91	0.85	0.8
Thr	0.83	0.73	0.66
Ca	0.90	0.84	0.76
Available P	0.45	0.42	0.38
Na	0.18	0.18	0.18
Cl	0.16	0.16	0.16

^1^ Providing the following amounts of vitamins and minerals per kilogram of a complete diet: vitamin A, 11,550 IU; vitamin D_3_, 4300 IU; vitamin E, 37.50 IU; vitamin K_3_, 3.85 mg; vitamin B_1_, 2.75 mg; vitamin B_2_, 9.90 mg; vitamin B_6_, 3.85 mg; vitamin B_12_, 22.00 mg; niacin, 49.50 mg; pantothenic acid, 15.40 mg; folic acid, 1.38 mg; biotin, 166.00 mg; selenium, 0.09 mg; iodine, 0.18 mg; copper, 3.00 mg; iron, 36.00 mg; manganese, 54.00 mg; zinc, 48.00 mg; cobalt, 0.12 mg.

**Table 2:** Effects of dietary *Ganoderma lucidum* powder supplementation on growth performance of broiler chickens.

	Experimental Treatment					
Items	Control	AGP	0.2% GLP	0.3% GLP	SEM	*p*-Values
1–8 days						
IBW (g)	48.20	48.05	48.50	48.03	0.682	0.181
BW (g)	234.52	237.27	237.95	235.12	1.130	0.604
FI (g)	222.25 ^a^	215.28 ^b^	213.04 ^b^	215.59 ^b^	1.779	0.019
FCR	1.19 ^a^	1.14 ^b^	1.12 ^b^	1.15 ^ab^	0.011	0.050
Viability (%)	100.00	100.00	100.00	100.00	−	−
9–18 days						
BW (g)	949.88	954.28	953.56	952.84	12.478	0.329
FI (g)	915.43 ^a^	915.72 ^a^	869.12 ^b^	908.04 ^ab^	8.425	0.017
FCR	1.28 ^a^	1.28 ^a^	1.22 ^b^	1.27 ^a^	0.016	0.025
Viability (%)	97.50	98.13	98.75	98.5	0.612	0.216
19–32 days						
BW (g)	2036.09 ^b^	2076.29 ^ab^	2121.37 ^a^	2045.12 ^b^	28.341	0.016
FI (g)	1864.46	1826.42	1864.31	1812.59	12.664	0.593
FCR	1.72 ^a^	1.63 ^b^	1.60 ^b^	1.66 ^ab^	0.032	0.007
Viability (%)	98.70	98.73	98.38	98.11	0.782	0.169
1–32 days						
FI (g)	3002.14 ^a^	2957.42 ^b^	2946.47 ^b^	2936.22 ^b^	8.293	0.019
FCR	1.51 ^a^	1.46 ^ab^	1.42 ^b^	1.47 ^ab^	0.017	0.037
Viability (%)	98.73	98.25	99.04	98.87	0.812	0.198

Data are expressed as means with their SEM. ^a,b^ Means without common letters within the same row differ at *p* < 0.05. Control, birds fed an antibiotic-free basal diet; AGP, birds fed a basal diet supplemented with antibiotic (0.02% zinc bacitracin); 0.2% GLP, birds fed a basal diet supplemented with 0.2% *G. lucidum* powder; 0.3% GLP, birds fed a basal diet supplemented with 0.3% *G. lucidum* powder. BW, body weight; FI, feed intake; FCR, feed conversion ratio.

**Table 3:** Effects of dietary *Ganoderma lucidum* powder supplementation on caecal microbiota of broiler chickens.

	Experimental Treatment					
Items	Control	AGP	0.2% GLP	0.3% GLP	SEM	*p*-Values
Total coliforms	6.06	6.01	6.12	5.80	0.160	0.904
*E. coli* (log CFU/g)	6.12	5.84	6.07	5.78	0.161	0.848
*Enterobacteriaceae* (log CFU/g)	6.18	6.25	6.29	6.03	0.140	0.919
pH	6.53	6.67	6.51	6.66	0.045	0.498
Fungi (log CFU/g)	2.85	2.56	3.31	3.27	0.135	0.191
Yeasts (log UFC/g)	4.36	4.45	4.07	3.80	0.160	0.489
LAB (log UFC/g)	5.55 ^b^	6.58 ^a^	6.63 ^a^	6.05 ^ab^	0.176	0.001

Data are expressed as means with their SEM. ^a,b^ Means without common letters within the same row differ at *p* < 0.05. Control, birds fed an antibiotic-free basal diet; AGP, birds fed a basal diet supplemented with antibiotic (0.02% zinc bacitracin); 0.2% GLP, birds fed a basal diet supplemented with 0.2% *G. lucidum* powder; 0.3% GLP, birds fed a basal diet supplemented with 0.3% *G. lucidum* powder. LAB, total lactic acid lactic bacteria.

**Table 4:** Effects of dietary *Ganoderma lucidum* powder supplementation on relative weights of immune organs of broiler chickens.

	Experimental Treatment					
Items	Control	AGP	0.2% GLP	0.3% GLP	SEM	*p*-Values
Thymus (%)	0.21 ^b^	0.30 ^a^	0.34 ^a^	0.32 ^a^	0.028	0.004
Bursa of Fabricius (%)	0.15	0.17	0.15	0.17	0.011	0.345
Spleen (%)	0.09	0.09	0.09	0.11	0.006	0.138

Data are expressed as means with their SEM. ^a,b^ Means without common letters within the same row differ at *p* < 0.05. Control, birds fed an antibiotic-free basal diet; AGP, birds fed a basal diet supplemented with antibiotic (0.02% zinc bacitracin); 0.2% GLP, birds fed a basal diet supplemented with 0.2% *G. lucidum* powder; 0.3% GLP, birds fed a basal diet supplemented with 0.3% *G. lucidum* powder.

**Table 5:** Effects of dietary *Ganoderma lucidum* powder supplementation on immunoglobulin concentrations of broilers.

	Experimental Treatment					
Items	Control	AGP	0.2% GLP	0.3% GLP	SEM	*p*-Values
IgG (mg/dL)	99.30	97.44	97.95	99.70	0.481	0.320
IgM (mg/dL)	180.47 ^a^	160.21 ^b^	170.45 ^ab^	181.71 ^a^	3.122	0.050
IgA (mg/dL)	73.81	72.75	74.78	76.49	1.355	0.242

Data are expressed as means with their SEM. ^a,b^ Means without common letters within the same row differ at *p* < 0.05. Control, birds fed an antibiotic-free basal diet; AGP, birds fed a basal diet supplemented with antibiotic (0.02% zinc bacitracin); 0.2% GLP, birds fed a basal diet supplemented with 0.2% *G. lucidum* powder; 0.3% GLP, birds fed a basal diet supplemented with 0.3% *G. lucidum* powder.

**Table 6:** Effects of dietary *Ganoderma lucidum* powder supplementation on mineral contents in the tibia of broilers.

	Experimental Treatment					
Items	Control	AGP	0.2% GLP	0.3% GLP	SEM	*p*-Values
Ash (%)	42.85 ^b^	42.18 ^b^	42.65 ^b^	45.34 ^a^	0.193	0.001
Ca (%)	11.20	11.52	11.33	12.28	0.273	0.327
P (%)	6.30	6.19	6.41	6.62	0.123	0.577

Data are expressed as means with their SEM. ^a,b^ Means without common letters within the same row differ at *p* < 0.05. Control, birds fed an antibiotic-free basal diet; AGP, birds fed a basal diet supplemented with antibiotic (0.02% zinc bacitracin); 0.2% GLP, birds fed a basal diet supplemented with 0.2% *G. lucidum* powder; 0.3% GLP, birds fed a basal diet supplemented with 0.3% *G. lucidum* powder.

## Data Availability

The data are available within this article.
